# Prevalence of increased myocardial extracellular volume fraction in dilated cardiomyopathy

**DOI:** 10.1186/1532-429X-15-S1-O31

**Published:** 2013-01-30

**Authors:** Magnus Lundin, Peder Sorensson, Andreas Fredholm, Anders Gabrielsen, Peter Kellman, Martin Ugander

**Affiliations:** 1 Department of Clinical Physiology, Karolinska Institutet, Stockholm, Sweden; 2 Department of Cardiology, Karolinska Institute, Stockholm, Sweden; 3 National Heart, Lung and Blood Institute, National Institutes of Health, Bethesda, MD, USA

## Background

Extracellular volume fraction cardiac magnetic resonance (ECV-CMR) enables quantitative myocardial tissue characterization. Increased myocardial ECV is correlated to myocardial fibrosis, inversely related to the left ventricle ejection fraction (LVEF), and an independent predictor of mortality. Dilated cardiomyopathy (DCM) is characterized by decreased LVEF and increased LV end-diastolic volume (EDV). We sought to determine the prevalence of increased myocardial ECV in DCM.

## Methods

Consecutive patients (n=213, mean age 51 years, range 13-84, 55% male) referred for clinical CMR evaluation of known or suspected heart disease were prospectively enrolled. CMR was undertaken at 1.5T using late gadolinium enhancement (LGE) and a Modified Look-Locker Inversion recovery (MOLLI) sequence. ECV images were generated using MOLLI-derived T1-mapping before and 15 minutes after an intravenous contrast bolus (gadoteric acid, 0.2 mmol/kg), and calibrated to hematocrit. Motion correction and co-registration was performed offline using an automated dedicated algorithm. ECV images were examined and focal and diffuse lesions were identified (normal ECV range 20-30%). If the ECV in remote, LGE-negative myocardium was >30% it was said to be diffusely increased. DCM was defined as LVEDVindex >100 ml/m2 and LVEF <58%. Data are presented as mean [range].

## Results

Out of 213 patients, 58 had DCM, among which 50/58 (85%) had normal myocardial ECV and 8/58 (14%) had diffusely increased myocardial ECV >30%. The patients in the diffusely increase myocardial ECV group were the same age (58 [13-75] vs 49 [17-81] years, years, p=0.24) and male to the same extent (75 vs 72%). Of the patients with normal myocardial ECV, 30/50 (60%) had focal lesions on LGE and 15/30 (50%) of these were ischemic lesions. Out of the patients with diffusely increased myocardial ECV, 3/8 (38%) had focal lesions, and one of these was an ischemic lesion. When compared to the DCM patients with normal ECV, the group with diffusely increased myocardial ECV did not differ in LVEDVindex (163 [120-289] vs 128 [101-212] ml/m2, p=0.18), had a lower LVEF (28 [12-42] vs 39 [13-58] %, p=0.02) and, as expected, higher myocardial ECV (35 [31-40] vs 25 [22-29] %, p<0.01).

## Conclusions

Our findings suggest that diffusely increased myocardial ECV can be found in approximately 1 in 7 patients with DCM. These patients with DCM and diffusely increased myocardial ECV had a more severe form of heart disease, manifested as lower LV systolic function compared to DCM patients with normal myocardial ECV.

## Funding

The study is supported (PI: MU) by the Swedish Research Council, Swedish Heart and Lung Foundation, Stockholm County Council, Swedish Society for Medical Research, Swedish Heart Assocation, Swedish Society of Medicine, Karolinska Institute, and the following foundations: Å. Wiberg, L.&H. Osterman, M. Kleberg, L.&J. Grönberg.

**Figure 1:**
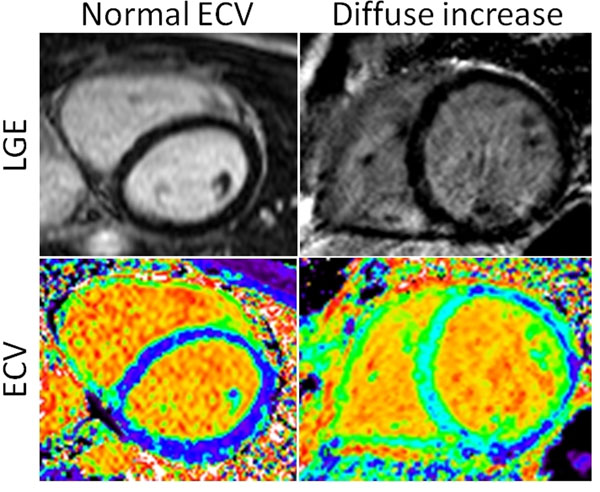
Representative LGE and ECV images of two patients with dilated cardiomyopathy (DCM), one with normal remote myocardium and one with diffusely increased myocardial extracellular volume fraction (ECV).

